# Transcriptional responses of *Leishmania* (*Leishmania*) *amazonensis* in the presence of trivalent sodium stibogluconate

**DOI:** 10.1186/s13071-019-3603-8

**Published:** 2019-07-12

**Authors:** Luz H. Patino, Carlos Muskus, Juan David Ramírez

**Affiliations:** 1 Grupo de Investigaciones Microbiológicas-UR (GIMUR), Programa de Biología, Facultad de Ciencias Naturales y Matemáticas, Universidad del Rosario, Bogotá, Colombia; 2 Programa de Estudio y Control de Enfermedades Tropicales (PECET), Facultad de Medicina, Universidad de Antioquia, Medellín, Colombia

**Keywords:** Resistance, Diffuse leishmaniasis, DEG, Hierarchical cluster analysis (HCA), Principal components analysis (PCA), Transcript

## Abstract

**Background:**

In the last decade, resistance to antimonials has become a serious problem due to the emergence of drug-resistant strains. Therefore, understanding the mechanisms used by *Leishmania* parasites to survive under drug pressure is essential, particularly for species of medical-veterinary importance such as *L. amazonensis*.

**Methods:**

Here, we used RNA-seq technology to analyse transcriptome profiles and identify global changes in gene expression between antimony-resistant and -sensitive *L. amazonensis* promastigotes.

**Results:**

A total of 723 differentially expressed genes were identified between resistant and sensitive lines. Comparative transcriptomic analysis revealed that genes encoding proteins involved in metabolism (fatty acids) and stress response, as well as those associated with antimony resistance in other *Leishmania* species, were upregulated in the antimony-resistant line. Most importantly, we observed upregulation of genes encoding autophagy proteins, suggesting that in the presence of trivalent stibogluconate (Sb^III^) *L. amazonensis* can activate these genes either as a survival strategy or to induce cell death, as has been observed in other parasites.

**Conclusions:**

This work identified global transcriptomic changes in an *in vitro*-adapted strain in response to Sb^III^. Our results provide relevant information to continue understanding the mechanism used by parasites of the subgenus *Leishmania* (*L. amazonensis*) to generate an antimony-resistant phenotype.

**Electronic supplementary material:**

The online version of this article (10.1186/s13071-019-3603-8) contains supplementary material, which is available to authorized users.

## Background

Leishmaniasis is a complex of tropical diseases caused by protozoan parasites of the genus *Leishmania*, characterised by a broad spectrum of clinical manifestations that have been classified into five categories: localised cutaneous leishmaniasis (CL); diffuse CL (DCL); disseminated CL (DL); mucocutaneous leishmaniasis (MCL); and visceral leishmaniasis (VL). Over 30 *Leishmania* species have been identified to date, and classified into four subgenera: *Leishmania* (*Leishmania*), *Leishmania* (*Viannia*), *Leishmania* (*Sauroleishmania*) and *Leishmania* (*Mundinia*) [1, 2]. Of the species belonging to subgenus *Leishmania*, *L. amazonensis* has particular clinical and epidemiological importance, especially in Latin America. *Leishmania amazonensis* is the main etiological agent of DCL, is implicated in borderline disseminated cutaneous leishmaniasis [3] and is responsible for 8% and 3% of CL cases in Brazil and Colombia, respectively [4, 5]. Additionally, several studies have identified *L. amazonensis* as a causative agent of VL in humans and animals (canines and felines), demonstrating its importance in both clinical and veterinary medicine [6–9].

To date, and in the absence of an available vaccine, chemotherapy is the only option for treatment of leishmaniasis. Although several different drugs are available, antimonials (e.g. sodium stibogluconate and meglumine antimoniate) remain standard treatment and the drugs of choice for treatment of all forms of leishmaniasis in different endemic areas (particularly Latin American). However, in the last decade there has been a large-scale increase in therapeutic failure of antimonials [10]. Although the incidence of therapeutic failure in patients infected with *L. amazonensis* is unclear, a percentage of patients who subsequently develop DCL (caused by a failure of the immune response) show a poor response to antimonials [11].

Numerous factors impact the final therapeutic outcome of antimonial treatment [12], with factors associated with the parasite itself. Several studies have focused on determining the mechanisms used by the parasite to survive under drug pressure using next-generation sequencing techniques [genomics, transcriptomics (RNA-seq), proteomics and metabolomics]. Some of these studies, mainly using strains of *L. donovani*, *L. major* and *L. infantum*, have demonstrated that, under drug pressure, *Leishmania* uses several adaptative mechanisms to modulate the gene dosage of therapeutic targets or other determinants of resistance. Some of these mechanisms include the generation of episomal amplicons, changes in ploidy of the whole chromosome and/or generation of local gene copy number variation, production of single-nucleotide polymorphisms in drug targets or upregulating proteins that may play a role in intracellular survival [13–18].

Recently, RNA-seq technology has emerged as a powerful tool in the study of *Leishmania* species. It has been used to determine the transcriptomic profiles of different species of *Leishmania* (*L. major*, *L. donovani*, *L. infantum*, *L. mexicana*, *L. amazonensis* and *L. braziliensis*), expanding our knowledge about parasite biology and their interactions with vertebrate and invertebrates hosts [19–23]. In addition, RNA-seq has been used to study the transcriptomic response to different stress conditions, and to identify genes associated with resistance to antimonials, mainly in strains of the *L. donovani* complex [15, 24, 25].

RNA-seq-based analyses have also been used in New World *Leishmania* species, including *L. amazonensis*, *L. braziliensis* and *L. mexicana*, to analyse transcriptional behaviour under specific conditions [20, 22, 26, 27]. However, none of these studies have focused on identifying transcriptional changes that occur in these parasites under stress conditions (such as drug pressure), as has been described for Old World *Leishmania* species. These data are particularly lacking for *L. amazonensis*, a species that is emerging as a pathogen of medical-veterinary importance in Latin America. Therefore, the purpose of this study was to conduct a comprehensive transcriptome profiling using RNA-seq to identify global changes in gene expression that occur in *L. amazonensis* in response to Sb^III^ exposure, and to obtain a general picture of the mode of action in which this species regulates *in vitro* gene expression under drug pressure. Our results contribute to the understanding of *in vitro* Sb^III^-resistance phenotypes and help to determine the global transcriptional effects of Sb^III^. This is also the first report providing transcriptome data for *L. amazonensis* submitted to a specific drug pressure.

## Methods

### Culture conditions and development of drug-resistant *L. amazonensis* promastigotes

Promastigotes of *L. amazonensis* [obtained from one patient with clinical CL symptoms from Medellin (Colombia) and named UA301] sensitive to Sb^III^ (Sb^III^-S) and resistant to Sb^III^ (Sb^III^-R) were axenically maintained in RPMI 1640 medium from Sigma-Aldrich (St. Louis, MO, USA) supplemented with 10% (v/v) heat inactivated fetal bovine serum from Thermo Fisher Scientific (Boston, MA, USA) and cultured at 26 °C with 5% CO_2_. DNA extraction and subsequent species identification, which was performed by direct Sanger sequencing of the cytochrome b (*cytb*) and heat-shock protein (*hsp70*) gene fragments, was carried out as described by Ramirez et al. [5].

The Sb^III^-resistant population, *L. amazonensis* (La-Sb^III^-R) promastigotes were obtained from wild-type sensitive *L. amazonensis* (La-Sb^III^-S) *via* the continuous stepwise increase in drug pressure with Sb^III^, as described previously [28], with slight modifications. The selection of resistant parasites was initiated in quadruplicates. Briefly, 10^6^ logarithmic-phase promastigotes were incubated with different concentrations of Sb^III^. The drug concentration was increased in a stepwise process only when the drug-exposed parasites had a growth rate similar to that of the parental parasites. Selection rounds were performed successively with 2-fold increase with 1.0, 2.0, 4.0, 8.0, 16, 32, 64 and 128 μg/ml Sb^III^. This incrementation was continued until the maximum concentration of parasite growth. After this period, the Sb^III^-R line was maintained for 3 weeks at the final drug concentration. To verify that the observed drug-resistant phenotype was stable, we cultivated the Sb^III^-resistant line for 4 weeks in the absence of Sb^III^. The Sb^III^-sensitive *L. amazonensis* was cultured in parallel, but without any drug pressure. At the end of this period, the susceptibility of the sensitive and resistant lines to Sb^III^ was determined by calculating the EC_50_ in an MTT [3-(4,5-dimethylthiazol-2-yl)-2,5-diphenyltetrazolium bromide] colorimetric assay, as previously described [29]. The reduction of MTT to its insoluble form formazan was evaluated in a Tecan GENios Microplate Reader (Biotek, Winooski, VT, USA), with an emission of 570 nm. The corresponding absorbance values were obtained from the spectrofluorometric reading and the EC_50_ was calculated using Graph Pad Prism v.5.0 software. The assays were performed three times in triplicate. Differences in the data were considered significant when the resistance index was ≥ 10-fold different between the Sb^III^-resistant and -sensitive lines. Once the parasites were selected (Sb^III^-resistant and sensitive), they were cloned from culture into 96-well plates containing RPMI medium supplemented, *via* limiting dilution as described previously [30].

### RNA isolation

Approximately 1 × 10^6^ promastigotes (sensitive and resistant to Sb^III^) in the middle logarithmic growth phase were cultured and harvested by centrifugation. The resulting pellets were used to conduct the RNA extraction. Total RNA was extracted from four independent replicates (two technical and two biological replicates) of each Sb^III^-resistant and -sensitive line, each originating from a separate culture. The RNA was extracted with the RNeasy Mini Kit (Qiagen, Hilden, Germany). The RNA concentrations were determined with a NanoDrop ND-1000 spectrophotometer (Thermo Fisher Scientific) and the quality and integrity with a 2100 Bioanalyzer system (Agilent Technologies, Santa Clara, CA, USA) according to the manufacturers’ instructions.

### Transcriptome sequencing and data analysis

The mRNA and cDNA library were prepared and sequenced with the HiSeq X-Ten system (Illumina, San Diego, CA, USA) by Novogene Bioinformatics Technology Co., Ltd, Beijing, China. Paired reads of 75 nucleotides were obtained for the mRNA libraries, whereas 2 × 100 bp length of reads were obtained for the cDNA libraries. Sequence quality metrics were assessed with FastQC (Illumina platform, PE 150, Q30 ≥ 80%; 250–300 bp insert cDNA library). Additionally, 20M raw reads/sample rRNA depletion was performed by poly(A) magnetic beads capture protocol, using Strand-specific TrueSeq RNA-seq Library Prep (Illumina), according to the manufacturer’s instruction.

Reads were mapped to the *L. mexicana* reference genome (MHOM/GT/2001/U1103) obtained from TriTrypDB (www.tritrypdb.org) using Smalt v.7.4 (http://www.sanger.ac.uk/science/tools/smalt-0). The *L. mexicana* genome was used as the *L. amazonensis* genome is not completely annotated. The amounts of each of the transcripts were quantified by assessing read depth, as described previously [31, 32]. For differential expression analysis, STAR v.2.5.2 was used for mapping and read counting per gene with default parameters where multiply mapped reads were marked and ignored. DEseq2 v.1.18.1 was then used to normalize the read counts and evaluate the statistical significance of differentially expressed genes. Here the following criteria were used: a fold-change cut-off of ≥ 2 and a Benjamini–Hochberg adjusted *P*-value < 0.05. The percentage of differentially expressed genes (DEGs) per chromosome was defined as follows: (number of differentially expressed genes per chromosome)/(number of total genes per chromosome) × 100.

In the initial data exploration, we constructed a principal components analysis (PCA) and hierarchical cluster analysis (HCA) to test whether both conditions (sensitive and resistant) could be clustered separately. The PCA was performed in R directly and was based on the variant stabilized count of each sample. The HCA was performed by applying the Euclidean distance measure and Ward’s algorithm. The Euclidean distance was calculated over the rlog-transformed count using DESeq2 and plotted using the *pHeatmap* R package (https://cran.r-project.org/). The four replicates of each condition (La-Sb^III^-S and La-Sb^III^-R) were used.

Gene Ontology enrichment analyses were performed using Tritrypdb tools (http://tritrypdb.org) with Fisher’s exact test used to maintain the FDR below 0.05. The GO terms were submitted to REVIGO, which is a web server that takes long lists of GO terms and summarizes them in categories and clusters of differentially expressed genes by removing redundant entries [33]. Finally, a Venn diagram was constructed using an online program provided by the Bioinformatics and Evolutionary Genomics group of the University of Gent and the VIB institute (http://bioinformatics.psb.ugent.be/webtools/Venn/).

## Results

### Induction of Sb^III^ resistance in *L. amazonensis* line

Initially, we selected *in vitro* populations of *L. amazonensis* that were resistant to Sb^III^. In the selection dynamics, two replicates did not survive; the third (4.0 µg/ml Sb^III^) and fourth (8.0 µg/ml Sb^III^) rounds of selection and two replicates were successfully selected to survive to seven rounds (64 μg/ml). At the highest Sb^III^ concentration (128 μg/ml), the parasites died (see Additional file 1: Figure S1). Likewise, when we evaluated the stability of the resistance phenotype (64 μg/ml Sb^III^ for 4 weeks), we observed that the resistance index of each line remained, suggesting that the *in vitro* selected drug resistance phenotype was stable.

### Differentially expressed transcripts between the Sb^III^-resistant and -sensitive *L. amazonensis* lines

**Fig. 1:**
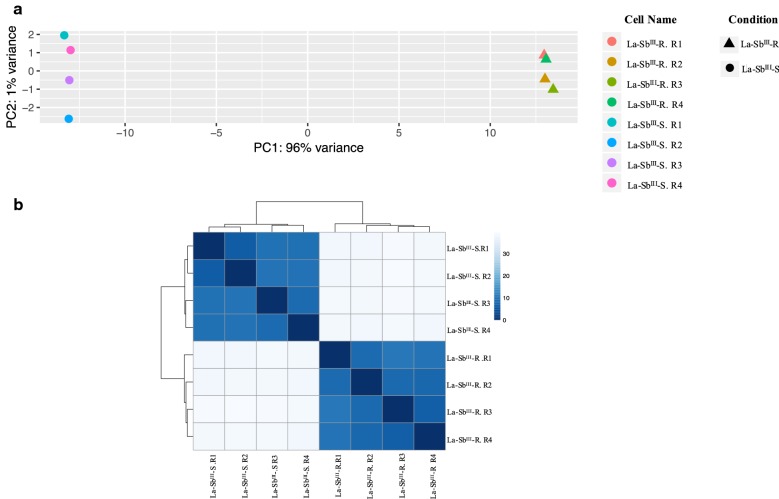
La-Sb^III^-S and La-Sb^III^-R lines discriminations. **a** Principal component analysis based on the variant stabilized counts from four individual replicates (La-Sb^III^-S and La-Sb^III^-R). **b** Hierarchical clustering analysis, which was based on data from four individual replicates (La-Sb^III^-S and La-Sb^III^-R lines) and plotted using the pHeatmap R package (https://cran.r-project.org/). Stronger relationships between variables are indicated by shorter distances in the dendrograms and darker blue color in the heatmap

As a first data exploration of the variation in our dataset, we performed a principal component analysis (PCA) and hierarchical cluster analysis (HCA). The results observed in the PCA showed that the first principal component explained 96% of the total variation in our experimental lines and clearly separated the La-Sb^III^-S from La-Sb^III^-R lines (Fig. 1a). Likewise, in the HCA, when Euclidean distance between samples was computed and used to create a heatmap colour image and dendrogram depicting the relatedness between samples, a clear separation between resistant and sensitive lines was observed (Fig. 1b).

**Fig. 2:**
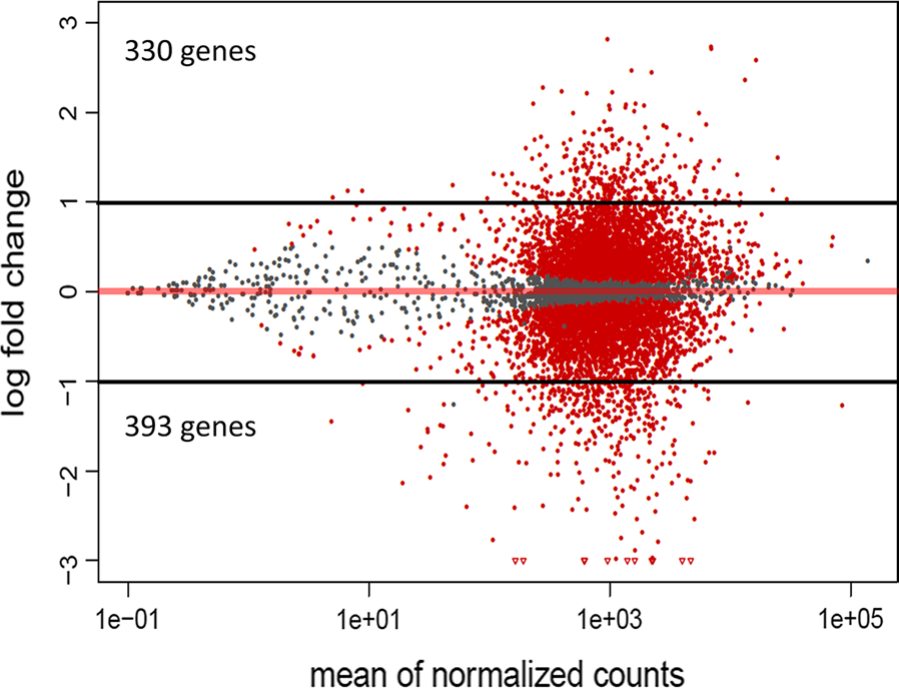
Graphical representation of genes differentially expressed between La-Sb^III^-S and La-Sb^III^-R. The figure represents the MA plot constructed based on the DESeq2 results, showing the relationship between mean expression (normalised counts) and fold-change for each gene. Each point represents one gene. Gray dots indicate the genes that were not differentially expressed and the red dots, located above and below of black discontinuous lines (cut-off for the fold-change (log fold-change > 1 and < − 1), represent differentially expressed genes with abs [log2(FC)] > 1 and an adjusted *P*-value < 0.01, between La-Sb^III^-S and La-Sb^III^-R

Later, we evaluated the expression profile of *L. amazonensis* under drug pressure, performing differential gene expression analysis of Sb^III^-sensitive and Sb^III^-resistant *L. amazonensis* lines (La-Sb^III^-S and La-Sb^III^-R, respectively). We identified a total of 723 genes that were differentially expressed between the two lines (*P*-value cut-off of < 0.05 and fold-change difference ≥ 2), 330 upregulated and 393 downregulated in the La-Sb^III^-R line (see Additional file 2: Table S1). Additionally, these genes were visualised using an MA plot showing the relationship between mean expression and fold-change for each gene (Fig. 2). Of the 723 genes that were significantly up/downregulated in the La-Sb^III^-R line, 46% (335/723) were annotated as hypothetical proteins, with the remaining gene products associated with various biological functions in the parasite (surface proteins, virulence, metabolism, cell cycle, autophagy, cytoskeletal and stress response).

### Gene Ontology (GO) and Kyoto Encyclopedia of Genes and Genomes (KEGG) enrichment analysis of differentially expressed genes (DEGs)

**Fig. 3:**
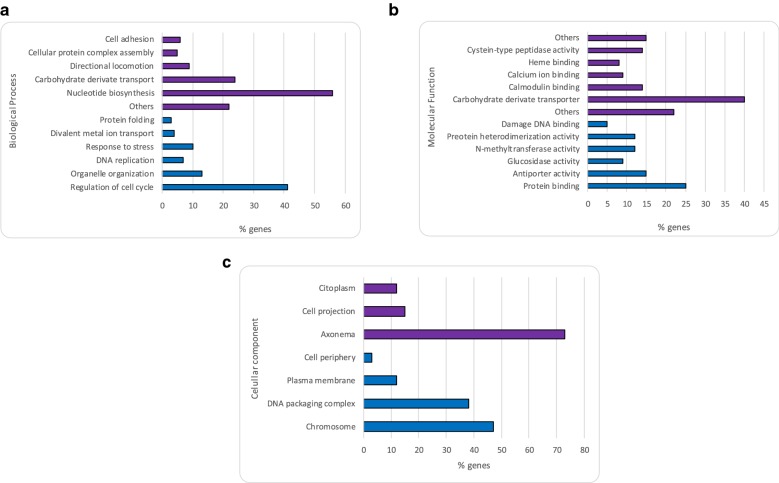
Gene Ontology (GO) predictions for the genes differentially expressed between La-Sb^III^-S and La-Sb^III^-R. The bar graphs show the most represented functions within three categories: biological process (**a**), molecular function (**b**) and cellular component (**c**). The blue and purple bars represent the up- and downregulation, respectively, of genes in the resistant line compared with the sensitive line

To better analyse the DEGs, we performed GO and KEGG enrichment-based analyses. The 723 DEGs were categorised into three functional GO groups: biological process; molecular function; and cellular component. Within the biological processes GO group, the genes upregulated in the La-Sb^III^-R line were mainly predicted to be involved in regulation of the cell cycle and organelle organisation but were also associated with stress response and divalent metal ion transport. However, the downregulated genes were involved in nucleotide biosynthesis and carbohydrate transport (Fig. 3a). For the molecular function group, genes upregulated in the La-Sb^III^-R line mainly encoded binding proteins and proteins with enzymatic activity, while the downregulated genes mainly encoded carbohydrate transporters and proteins with peptidase activity (Fig. 3b). Finally, within the cellular components group, the up- and downregulated genes in the La-Sb^III^-R line encoded proteins localised mainly in the nuclear component and in the axoneme, respectively (Fig. 3c).

**Fig. 4:**
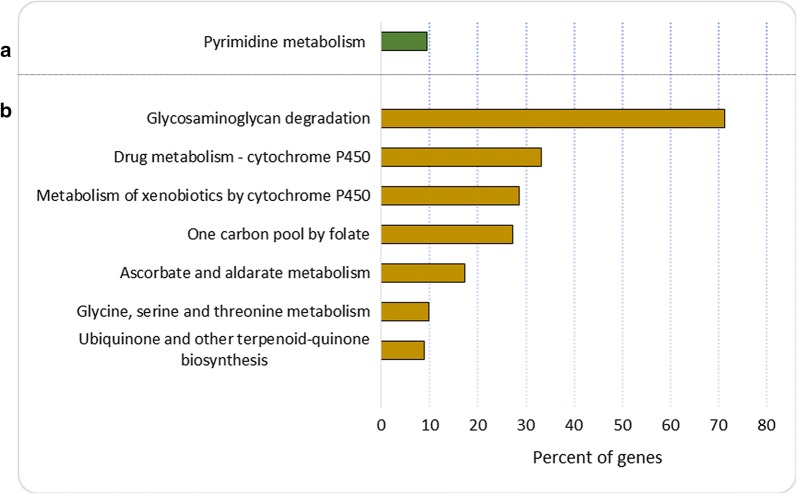
Kyoto Encyclopedia of Genes and Genomes enrichment analysis for the genes differentially expressed between La-Sb^III^-S and La-Sb^III^-R. The bar graphs show the pathways regulated by upregulated (**a**) and downregulated (**b**) genes in the La-Sb^III^-R line compared with the La-Sb^III^-S line

KEGG enrichment analysis revealed that genes upregulated in the La-Sb^III^-R line were involved in pyrimidine metabolism, while the downregulated genes were involved in ubiquinone biosynthesis, glycine, serine and threonine metabolism, ascorbate and aldarate metabolism, drug metabolism-cytochrome P450 and glycosaminoglycan degradation (Fig. 4, Table 1).

**Table 1:** KEGG enrichment analysis of the up- and downregulated genes in the La-Sb^III^-R line

Pathway ID	Map name	Genes
Upregulated genes		
ec00240	Pyrimidine metabolism	LmxM.13.1630, LmxM.18.1580, LmxM.21.1210, LmxM.23.0680, LmxM.28.0890, LmxM.28.1420, LmxM.28.1430, LmxM.33.0010, LmxM.34.1790
Downregulated genes		
ec00130	Ubiquinone and other terpenoid-quinone biosynthesis	LmxM.05.1100, LmxM.34.0500, LmxM.34.0500a, LmxM.34.0520b, LmxM.34.0540, LmxM.34.0550
ec00260	Glycine, serine and threonine metabolism	LmxM.10.0090, LmxM.14.1320, LmxM.29.2090, LmxM.32.0520, LmxM.36.3800
ec00053	Ascorbate and aldarate metabolism	LmxM.10.0090, LmxM.18.0160, LmxM.26.0160, LmxM.32.0520, LmxM.33.0010, LmxM.34.0500, LmxM.34.0500a, LmxM.34.0520b, LmxM.34.0540, LmxM.34.0550
ec00670	One carbon pool by folate	LmxM.14.1320, LmxM.36.3800, LmxM.36.6390
ec00980	Metabolism of xenobiotics by cytochrome P450	LmxM.29.2090, LmxM.32.0240
ec00982	Drug metabolism - cytochrome P450	LmxM.29.2090, LmxM.32.0240
ec00531	Glycosaminoglycan degradation	LmxM.34.0500, LmxM.34.0500a, LmxM.34.0520b, LmxM.34.0540, LmxM.34.0550

### Surface molecules

Thirteen transcripts encoding surface proteins were downregulated in the La-Sb^III^-R line compared with the La-Sb^III^-S line. Eight of these encoded surface antigen-like protein (PSA), four were expressed in tandem in the chromosome 4, two encoded proteophosphoglycan ppg3/ppg1 and the remaining transcripts encoded lipophosphoglycan (LPG), surface membrane protein gp46-like and major surface protease gp63 (GP63, or leishmanolysin). The most strongly downregulated transcripts in the La-Sb^III^-R line were homologous transcript described in *L. mexicana*, a close species related with *L. amazonensis*, LmxM.05.0900, LmxM.34.0500 and LmxM.28.0570, encoding PSA, proteophosphoglycan ppg3 and major surface protease gp63, respectively (Table 2). Despite mainly observing downregulation of surface molecules in the La-Sb^III^-R line, five transcripts (LmxM.08.0720, LmxM.08.0730, LmxM.08.0740, LmxM.28.1400 and LmxM.33.1920) encoding amastin-like surface protein were upregulated. Three of these transcripts were expressed in tandem from chromosome 8.

**Table 2:** List of most highly differentially-expressed genes between the La-Sb^III^-S and La-Sb^III^-R lines (*P*-value cut-off < 0.05 and fold-change difference ≥ 2)

Biological functions	Transcript	Product description	Log_2_ fold-change (R/S)
Surface proteins	LmxM.09.0580	Surface antigen-like protein	− 1.1603
	LmxM.05.1215	Surface antigen-like protein	− 1.2685
	LmxM.21.1170	Surface antigen-like protein	− 1.2882
	LmxM.04.0190	Surface antigen-like protein	− 1.8081
	LmxM.04.0210	Surface antigen-like protein	− 1.9894
	LmxM.04.0180	Surface antigen-like protein	− 2.0656
	LmxM.04.0200	Surface antigen-like protein	− 2.3355
	LmxM.05.0900	Surface antigen-like protein	− 3.7896
	LmxM.34.0550	Proteophosphoglycan ppg1	− 1.5215
	LmxM.34.0500	Proteophosphoglycan ppg3, putative	− 3.2111
	LmxM.33.3120	Lipophosphoglycan biosynthetic protein (lpg2)	− 1.2312
	LmxM.30.1450	Surface membrane protein gp46-like protein	− 1.3431
	LmxM.28.0570	Major surface protease gp63, putative	− 3.0596
	LmxM.08.0730	Amastin-like protein, putative	1.0151
	LmxM.28.1400	Amastin-like protein	1.1033
	LmxM.08.0720	Amastin-like protein, putative	1.1165
	LmxM.08.0740	Amastin-like protein, putative	1.2047
	LmxM.33.1920	Amastin-like surface protein, putative	1.7594
Metabolism	Metabolite transporters		
	LmxM.10.0350	Pteridine transporter ft5, putative	− 1.0251
	LmxM.24.0360	UDP-galactose transporter	− 1.0742
	LmxM.15.1230	Nucleoside transporter 1, putative	− 1.4466
	LmxM.32.0290	Glucose transporter/membrane transporter D2, putative	− 1.5513
	LmxM.15.1240	Nucleoside transporter 1, putative	− 1.9139
	LmxM.30.0320	Amino acid transporter, putative	− 1.9548
	Glycolysis		
	LmxM.10.0510	Glycerol-3-phosphate dehydrogenase [NAD+], glycosomal/mitochondrial	− 1.1530
	Proteolysis		
	LmxM.08.1080	Cathepsin L-like protease, putative	− 2.2280
	Fatty acid pathways		
	LmxM.30.2970	Acetyl-CoA carboxylase	1.0198
	LmxM.23.0710	Acetyl-CoA synthetase, putative	1.3180
	Other metabolic enzymes		
	LmxM.29.1940	Succinyl-coa:3-ketoacid-coenzyme a transferase- like protein	− 1.5541
	LmxM.26.1610	Proline dehydrogenase, mitochondrial	1.2112
	LmxM.27.0880	2-oxoglutarate dehydrogenase subunit, putative	1.1954
Cell cycle	LmxM.20.0030	Histone-lysine N-methyltransferase, H3 lysine-76 specific	2.4946
	LmxM.07.0025	Histone-lysine N-methyltransferase, putative	2.4788
	LmxM.10.0990	Histone H3	2.0457
	LmxM.10.0870	Histone H3	1.8415
	LmxM.25.0920	Histone RNA hairpin-binding protein	1.5715
	LmxM.20.0050	Histone chaperone ASF1A	1.3835
	LmxM.36.0020	Histone H4	1.2945
	LmxM.19.0050	Histone H2B	1.2791
	LmxM.30.3180	Histone H4	1.1477
	LmxM.34.1310	Histone H4	1.0938
	LmxM.19.0030	Histone H2B	1.0316
	LmxM.25.1470	Cyclin	2.3643
	LmxM.31.3320	Cyclin 6, putative	1.2431
	LmxM.28.1420	DNA polymerase kappa, putative	1.2764
	LmxM.28.1430	DNA polymerase kappa, putative	1.2214
	LmxM.34.1790	DNA polymerase epsilon subunit B, putative	1.1082
	LmxM.13.1630	Mitochondrial DNA polymerase I protein D, putative	1.0411
Autophagy	LmxM.27.0390	Autophagy protein APG9, putative	1.2217
	LmxM.23.1170	Membrane-bound acid phosphatase 2	1.5534
Cytoskeletal	LmxM.08.1230	β-tubulin	− 1.1278
	LmxM.21.1860	β-tubulin	− 1.8051
	LmxM.32.0792	β-tubulin	− 1.9300
	LmxM.05.0040	Paraflagellar rod component par4, putative	− 1.2326
	LmxM.16.1430	Paraflagellar rod protein 2	− 1.2388
	LmxM.09.1320	Paraflagellar rod component, putative	− 1.3933
	LmxM.08_29.1750	Paraflagellar rod protein 1D, putative	− 1.3995
	LmxM.36.4780	Paraflagellar rod component, putative	− 1.6234
	LmxM.16.1425	Paraflagellar rod protein 2C	− 1.9274
	LmxM.08_29.1760	Paraflagellar rod protein 1D, putative	− 1.9294
	LmxM.07.0310	Paraflagellar rod protein, putative	− 2.0882
	LmxM.32.0610	Paraflagellar rod component, putative	− 2.3516
	LmxM.36.4230	Paraflagellar rod component, putative	− 3.0082
Transporters associated with antimony response	LmxM.28.1930	Zinc transporter 3, putative	1.5220
	LmxM.30.3070	Ferrous iron transport protein	1.1716
	LmxM.23.0250	ABC-thiol transporter	1.1165
	LmxM.19.0180	Mitogen-activated protein kinase 9, putative	1.0732
	LmxM.13.0440	Mitogen-activated protein kinase kinase 2	1.0641
	LmxM.30.1290	Multidrug-resistance protein, copy 1-like protein	1.1374
H-locus	LmxM.23.0230	Hypothetical protein, conserved	1.0664
	LmxM.23.0240	Terbinafine resistance locus protein (yip1)	1.3429
	LmxM.23.0250	ABC-thiol transporter	1.1165
	LmxM.23.0260	Argininosuccinate synthase, putative	1.2138
Chaperones and stress proteins	LmxM.36.2030	Chaperonin HSP60, mitochondrial precursor	1.1311
	LmxM.28.2780	Heat-shock protein hsp70, putative	2.6066
	LmxM.32.0312	Heat-shock protein 83-1	2.3109
	LmxM.32.0316	Heat-shock protein 83-1	2.1008
	LmxM.32.0314	Heat-shock protein 83-1	2.0412
	LmxM.18.1370	Heat-shock protein, putative	1.3501
	LmxM.28.2770	Heat-shock protein hsp70, putative	1.1175

### Metabolism

We then analysed and compared the expression of transcripts associated with the transport of sugar, nucleobases and amino acids between the La-Sb^III^-S and La-Sb^III^-R lines. Most of the transcripts associated with these processes were downregulated in the resistant line, including six transcripts (LmxM.10.0350, LmxM.24.0360, LmxM.15.1230, LmxM.32.0290, LmxM.15.1240 and LmxM.30.0320) annotated as coding for a pteridine transporter, a UDP-galactose transporter, a nucleoside transporter 1, a glucose transporter/membrane transporter D2, a nucleoside transporter 1 and an amino acid transporter, respectively.

Several genes encoding proteins associated with various metabolic pathways (glycolytic pathway, tricarboxylic acid cycle and proteolysis) were also differentially expressed between the sensitive and resistant lines. Downregulated genes in the La-Sb^III^-R line included LmxM.10.0510, encoding a glycerol-3-phosphate dehydrogenase [NAD+] enzyme that not only catalyses the interconversion of dihydroxyacetone phosphate and l-glycerol-3-phosphate during the glycolysis, but also is important in both lipid and carbohydrate metabolism; LmxM.08.1080, encoding cathepsin-L protease, which is putatively involved in proteolysis; and LmxM.29.1940, encoding succinyl-CoA:3-ketoacid-coenzyme, a transferase involved in the catabolism of ketone bodies. These three transcripts were approximately 1.1-fold, 2.2-fold and 1.5-fold less abundant, respectively, in La-Sb^III^-R than in susceptible line La-Sb^III^-S.

Analysis of genes involved in the fatty acids pathway, which is associated with the intracellular amastigote stage and with promastigotes in culture, revealed upregulation in the La-Sb^III^-R line, of transcripts encoding a putative acetyl-CoA carboxylase (LmxM.30.2970), a putative acetyl-CoA synthetase (LmxM.23.0710), a mitochondrial proline dehydrogenase (LmxM.26.1610) and a 2-oxoglutarate dehydrogenase subunit (LmxM.27.0880), which is associated with the TCA cycle (Table 2).

### Cell cycle and autophagy

Among the upregulated cell cycle-associated genes in the La-Sb^III^-R line, histone protein-coding genes were particularly enriched. Eleven transcripts (LmxM.10.0990, LmxM.10.0870, LmxM.36.0020, LmxM.19.0050, LmxM.30.3180, LmxM.34.1310, LmxM.19.0030, LmxM.20.0030, LmxM.07.0025, LmxM.25.0920 and LmxM.20.0050) encoding histone-family or associated proteins, including histone 3, histone 4 and histone 2B, were upregulated in the resistant line. Additionally, transcripts coding for proteins related to cellular replication were also upregulated in the La-Sb^III^-R line, including those encoding cyclin and cyclin 6 (LmxM.25.1470 and LmxM.31.3320), along with various polymerases (LmxM.28.1420, LmxM.28.1430, LmxM.34.1790 and LmxM.13.1630), such as DNA polymerase kappa, DNA polymerase epsilon subunit B and mitochondrial DNA polymerase I protein D.

We also observed upregulation of transcripts encoding autophagy protein APG9 (LmxM.27.0390) and membrane-bound acid phosphatase 2 (MBAP2) (LmxM.23.1170) in La-Sb^III^-R, both of which have been associated with the recycling of proteins under stress conditions and/or while undergoing a differentiation process (Table 2).

### Cytoskeleton

Our analysis also identified differential expression of transcripts encoding proteins associated with the cytoskeleton between the La-Sb^III^-R and La-Sb^III^-S lines. We observed that three transcripts encoding β-tubulin and 10 transcripts encoding paraflagellar rod protein 1D were between 1.2-fold and 3.0-fold less abundant in the La-Sb^III^-R line than in susceptible line La-Sb^III^-S (Table 2).

### Antimonial resistance and stress response

Some of the genes previously associated with antimonial resistance mechanisms in *Leishmania* species were shown to be differentially expressed between La-Sb^III^-R and La-Sb^III^-S. In La-Sb^III^-R, upregulated genes included LmxM.28.1930 (zinc transporter 3), LmxM.30.3070 (ferrous iron transport protein), LmxM.23.0250 (ABC-thiol transporter), LmxM.19.0180 and LmxM.13.0440 (mitogen-activated protein kinase 9/2) and LmxM.30.1290 [multidrug resistance protein, copy 1-like protein (MDR1)]. In addition, amplicons derived from the H locus were also upregulated in La-Sb^III^-R, including transcripts coding for a hypothetical protein (LmxM.23.0230), HTB or terbinafine-resistance locus protein (Yip1) (LmxM.23.0240), an ABC-thiol transporter (MRPA) (LmxM.23.0250) and a putative argininosuccinate synthase (LmxM.23.0260).

Genes encoding several heat-shock proteins of different molecular masses were also upregulated in the La-Sb^III^-R line. Seven transcripts coding for heat-shock protein family members HSP70, HSP83-1 and HSP60 (LmxM.28.2780, LmxM.32.0312, LmxM.32.0316, LmxM.32.0314, LmxM.18.1370, LmxM.28.2770 and LmxM.36.2030) were approximately 2-fold more abundant in the resistant line (Table 2).

## Discussion

RNA-seq technology was used to characterise alterations in gene expression of *L. amazonensis* resulting from experimental induction of Sb^III^ resistance compared with an uninduced strain*. Leishmania amazonensis* is tremendously important in public health terms in Brazil and Colombia because of its association with CL and, more recently, VL in both humans and domestic animals (cats and dogs) [34, 35]. This association not only indicates the severity of *L. amazonensis* infection, but also the possible emergence of a domestic cycle and an increased risk of disease transmission. Until now, different approaches have been used with the purpose of understanding the transcriptomic behaviour of different species of *Leishmania* against antimonials; however, to our knowledge, this is the first attempt to elucidate and demonstrate the global gene expression profile of *L. amazonensis* under Sb^III^ pressure through RNA-seq. Herein, we identified a large number of genes showing differential expression between the sensitive and resistant lines (Fig. 2). Among these were transcripts encoding proteins associated with various biological processes, including adhesion, metabolism, cell cycle, autophagy, structural organisation and stress response (Fig. 3a).

Transcriptomic analysis of the different membrane-related proteins revealed differences between the La-Sb^III^-S and La-Sb^III^-R lines. Five transcripts encoding amastin proteins were overexpressed in La-Sb^III^-R (Table 2). The amastins are surface glycoproteins whose expression has been noted in other parasites such as *Trypanosoma cruzi* and *Trypanosoma brucei* (amastigotes and epimastigotes) [36, 37], as well as in two related insect parasites, *Leptomonas seymouri* and *Crithidia* spp. [38] and had been involved in host-parasite interactions, with roles in both infection and survival [38]. The upregulation of genes encoding amastin in our resistant line is consistent with a previous report [15], and although the relationship between this surface protein and antimonial resistance has not previously been demonstrated in *Leishmania*, our results suggest that overexpression of genes encoding amastin could increase the resistance of the parasite to the cellular stresses elicited by Sb^III^. In contrast, other surface protein-encoding genes, including those coding for PSA, proteophosphoglycan ppg3/ppg1, LPG, surface membrane protein gp46-like protein and major surface protease gp63/leishmanolysin, appeared to be downregulated in the resistant line (Fig. 3a, Table 2). Of these, only GP63 has previously been identified on the surface of *Leishmania* and other trypanosomatid species [39]. The downregulation of these genes under our study conditions suggests that *L. amazonensis* reduces the expression of some genes involved in virulence, interaction and survival in macrophages that are not necessary for survival under drug pressure. Future studies are needed in insect cell lines/macrophages to determine whether these genes are also downregulated during the *Leishmania* infection process.

On the other hand, most trypanosomatid species predominantly utilise glycolysis, amino acid metabolism and the fatty acid pathway (promastigotes maintained in culture) for energy generation [40–42]. Previous studies in *Leishmania* species have suggested that antimonials not only alter energetic metabolism by inhibiting glycolysis and fatty acids oxidation [10], but also cause changes in the transport of nutrients through the plasma membrane, as has been observed in Sb-resistant *Leishmania* strains [43]. Although we did not observe large variations in the expression of genes associated with metabolism between the sensitive and resistant lines, changes in the expression of genes encoding proteins associated with the glycolytic pathway or encoding glycolytic enzymes essentials in both lipid and carbohydrate metabolism and ATP production (downregulation of glucose transporter/membrane transporter D2 and glycerol-3-phosphate dehydrogenase [NAD+]) were consistent with previous reports in Sb-resistant *L. amazonensis* [44, 45] (Table 2). Additionally, GO analysis revealed a strong downregulation of genes involved in carbohydrate transport (Fig. 3b), which suggests decreased formation of reactive oxygen species as a result of reduced glucose uptake, thereby aiding survival in the oxidative environment triggered by the drug [45].

In the present study, we observed the upregulation of 11 transcripts in the Sb^III^-resistant line encoding histone proteins, namely H2B, H3 and H4 (Table 2). These proteins are associated with various biological processes in *Leishmania* and other trypanosomatids (*T. brucei* and *T. cruzi*) and are closely associated with transcription, DNA replication, recombination and repair [46–49], and likewise have been associated with antimony resistance in *Leishmania* parasites [15, 50]. GO analysis also confirmed a strong upregulation of genes involved in the regulation of the cell cycle (Fig. 3b), which agrees with data presented in a previous report [50]. These results reinforce the previously-noted association of histone proteins with resistance to antimonials found mainly in *L. donovani* [18, 50], and suggest similar behaviour in New World *Leishmania* species such as *L. amazonensis*.

Previous studies showed that the recycling of proteins by autophagic mechanisms is associated with metabolism in cells that are undergoing a differentiation process (metacyclogenesis) and/or under stress conditions [51, 52]. Our study identified upregulation of mRNA from chromosome 27 corresponding to the putative APG9 protein (Table 2), which is involved in autophagy and cytoplasm-to-vacuole transport (Cvt) vesicle formation, in the La-Sb^III^-R line. This suggests that in the presence of Sb^III^, *L. amazonensis* activates genes that induce autophagy, either as a survival strategy or as a form of cell death. This has also been observed in other parasites such as *T. brucei*, *T. cruzi*, *Leishmania donovani*, *Toxoplasma gondii* and *Plasmodium falciparum*, which activate different autophagy proteins (ATG3, ATG5, ATG7, ATG24 and PI3K) during nutrient starvation and under drug-induced stress as a mechanism of programmed cell death [53–55].

Another factor that may trigger protein recycling is purine starvation. *Leishmania*, *Trypanosoma* and *Toxoplasma* do not synthesise purines *de novo* and must scavenge them from the environment [56–58]. In response to this starvation, alterations are made to different metabolic processes, such as upregulation of purine salvage machinery. One of the most upregulated genes in purine-starved *Leishmania* parasites codes for membrane-bound acid phosphatase (MBAP2), which has a role in endosomal trafficking [52]. In the present study, we observed upregulation of the MBAP2 transcript in the La-Sb^III^-R line (Table 2), suggesting an increase in lysosome-related recycling processes, as has been noted in *L. major* [52].

Additionally, studies have demonstrated that drug pressure produces changes at the cytoskeletal level (α- and β-tubulin proteins), provoking several mutations related to drug resistance. This phenomenon has been identified in *Leishmania* species, including *L. tarentolae* [59], and has also been present in the homologous genes from *T. cruzi*, *T. brucei* and *T. evansi* [18, 60]. In the present study, we observed downregulation of transcripts encoding β-tubulin and paraflagellar rod protein 1D in the Sb^III^-resistant line (Table 2), as was recently observed in a resistant strain of *L. braziliensis* [61]. These results suggest that the development of antimony resistance may cause changes in cytoskeleton proteins as well.

Finally, several studies support the existence of a variety of resistance mechanisms in *Leishmania* parasites. One known mechanism of antimony resistance involves the reduction of drug accumulation by either reduced uptake or increased efflux through different membrane transporters, the most studied of which belongs to the ATP-binding cassette (ABC) protein superfamily [16, 62]. These protein transporters have been identified in other parasites including *T. brucei* and *T. cruzi*, and as in *Leishmania* species, their overexpression is implicated in resistance to different drugs [63–65]. In the present transcriptomic analysis, we observed upregulation of different transcripts encoding protein transporters in the La-Sb^III^-R line (Table 2), all of which have previously been implicated in resistance to antimonials in other *Leishmania* species [15, 16]. These transporters included zinc transporter 3, ferrous iron transport protein and membrane transporters of the ABC superfamily (MDR1 and MRPA).

The *L. amazonensis* mdr1 gene, which has demonstrated to be 91 and 78% identical to the closely related ldmdr1 gene in *L. donovani* and lemdr1 gene in *L. enriettii*, respectively [66, 67], has been shown to be overexpressed in amphotericin B- and Sb-resistant strains of *L. donovani* [68–70], in a melarsoprol-resistant strain of *T. brucei* [71, 72] and in benznidazole-resistant epimastigotes of *T. cruzi* [64, 65]. Otherwise the gene encoding MRPA, which is one of three genes related to drug resistance identified within the H locus and which is amplified in extrachromosomal circles of DNA, was overexpressed in a number of *Leishmania* strains selected for resistance to Sb^III^, Sb^V^ or the related metal [15, 73–76]. Additionally, overexpression of MRPA has been reported to decrease the influx of antimony rather than increase efflux [10]. The overexpression of genes that encode the MDR1 and MRPA transporters in our experimentally-induced Sb^III^-resistant *L. amazonensis* strain suggests that active efflux/influx of Sb^III^ is a mechanism used by this species to survive in the presence of drug pressure, supporting previous reports in other species.

We also observed upregulation of genes coding for mitogen-activated protein kinases (MAPKs), which have been associated with important cell processes such as proliferation, differentiation, cell shape, stress response, apoptosis and immune evasion in trypanosomatids [77, 78], and putatively with antimony resistance in *Leishmania* parasites [79]. Of the 17 MAPKs and MAPK-like kinases identified in *Leishmania* [80], only MAPK1 has previously been associated with antimony resistance. However, expression of the MAPK1 gene in resistant *L. donovani* appears variable, with some reports showing consistent upregulation in resistant isolates [50] and others showing downregulation in antimony-resistant field isolates [79, 81]. Although genes coding for MAPK2 and MAPK9 were upregulated in our resistant line, neither of these proteins have previously been reported in Sb^III^-resistant strains, suggesting that their association with antimony resistance should be further studied.

Other genes overexpressed in the resistant *L. amazonensis* line were those encoding heat-shock proteins (HSPs). HSPs are a family of proteins whose function is to protect the cell from toxic external stimuli. Various *in vitro* studies have recorded the overexpression of different HSPs in drug-resistant *Leishmania* strains [15, 18, 82, 83]. However, although HSPs are the most abundant proteins in *T. cruzi* [84], their role in drug resistance remains unclear [85]. Of the HSPs identified in *Leishmania* parasites, HSP83 and HSP70 are involved in the activation of programmed cell death mediated by drugs, as they interfere with the mitochondrial membrane potential as has been observed in strains of *L. donovani* [83, 86]. In the present study, we observed the overexpression of transcripts encoding HSP70, HSP83 and HSP60 in the La-Sb^III^-R line (Table 2). This supports previous findings [61] and reinforces the role of these proteins in resistance to antimony, both in Old and New World *Leishmania* species.

## Conclusions

The transcriptomic analysis conducted in this study identified several transcripts that were differentially abundant between the antimony-resistant and -sensitive lines, several of which have previously been reported as potential therapeutic targets in Old World species as well as some New World species, including *L. braziliensis*, *L. guyanensis* and *L. panamensis.* Thus, we conclude that next-generation sequencing technologies are, and will continue to be, the gold standard techniques for understanding transcriptomic behaviour of a large number of organisms, increasing our knowledge of poorly understood species. Finally, although various studies propose intracellular amastigotes as the gold standard for *in vitro Leishmania* drug discovery research and evaluation of resistance [87, 88], we focused our molecular analysis on the promastigote stage for several reasons: the amastigote model is (i) time-consuming, (ii) laborious, (iii) difficult to manipulate in terms of inducing Sb^III^-resistance [89, 90], and (iv) difficult to scale, thereby limiting its use in high-throughput screening approaches [91]. However, considering that the amastigote stage is the infectious form in the host, and that some of the genes with differential expression found in this study have been previously described by other researches using axenic amastigotes [22, 26], the results obtained here can be used in the future to guide targeted studies in this parasite infective stage. Future studies need to be conducted to validate the transcriptomic responses herein described.

## Additional files

**Additional file 1: Figure S1.** Percent viability (Y-axis) of *L. amazonensis* promastigotes treated for 72 h with different concentrations of Sb^III^ (1.0 to 128.5 µg/ml), represented as [Sb^III^] Log_10_ (X-axis). The arrows show the IC50 reached by each line.

**Additional file 2: Table S1.** List of differentially expressed genes between La-SbIII-S and La-SbIII-R lines with a fold-change ≥ 2.

## Data Availability

Data supporting the conclusions of this article are included within the article and its additional files. The dataset generated during the present study was deposited at DDBJ/ENA/GenBank under the accession number PRJEB31417.

## Abbreviations

La: *Leishmania amazonensis*
Sb^III^: trivalent sodium stibogluconate
DEG: differentially expressed gene
HSP: heat-shock protein
RNA-seq: ribonucleic acid sequencing
