# Supplementary figures and images for: Transcriptional responses of Leishmania (Leishmania) amazonensis in the presence of trivalent sodium stibogluconate
Source: Parasit Vectors. 2019 Jul 12;12:348. doi: 10.1186/s13071-019-3603-8 (PMC6626383; doi:10.1186/s13071-019-3603-8)

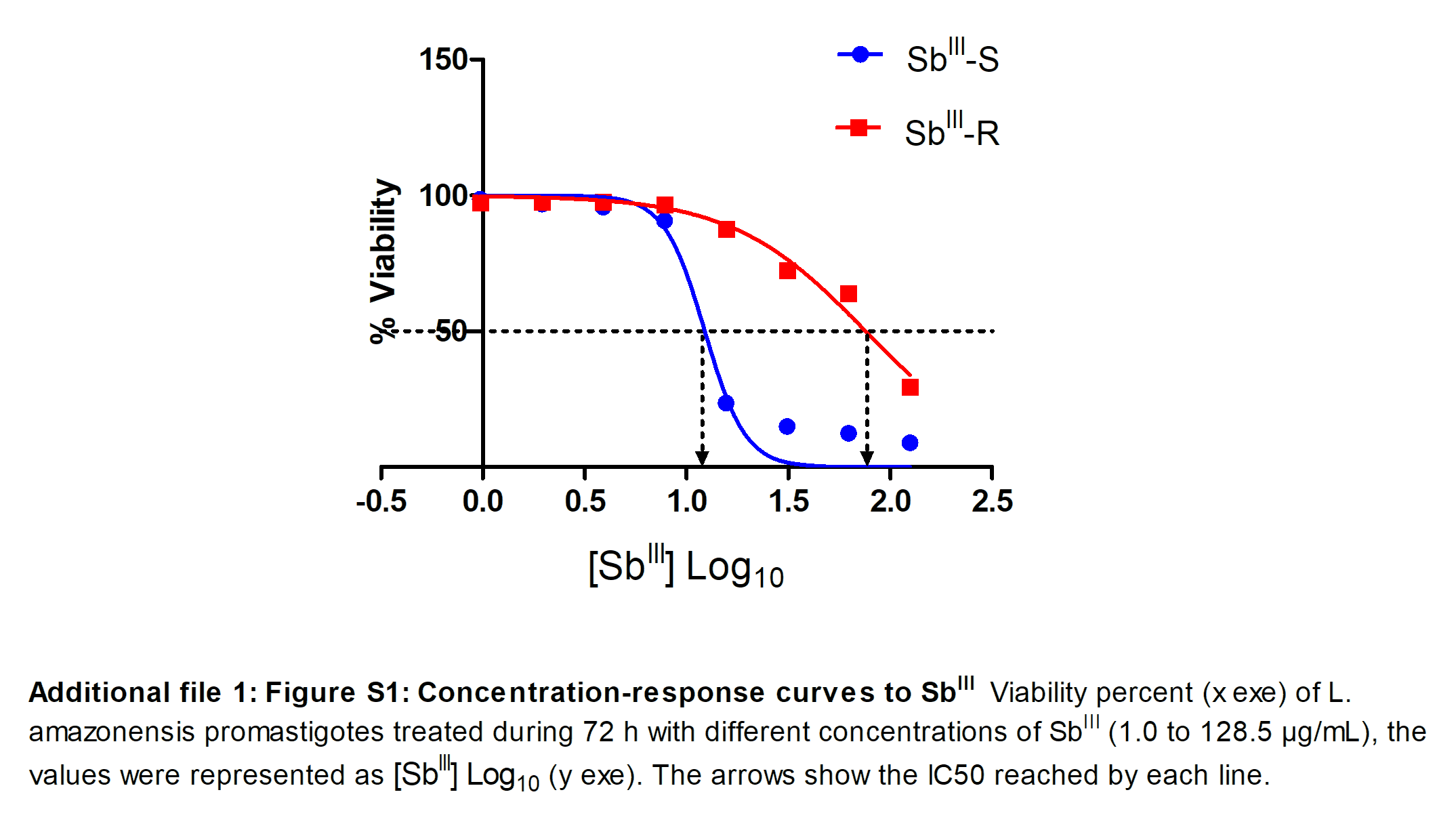

Supplement: Supplementary file 1 — Additional file 1: Figure S1. Percent viability (Y-axis) of L. amazonensis promastigotes treated for 72 h with different concentrations of SbIII (1.0 to 128.5 µg/ml), represented as [SbIII] Log10 (X-axis). The arrows show the IC50 reached by each line. [file 13071_2019_3603_MOESM1_ESM.tif]
